# Chronic conditions and adolescent psychosocial functioning: the moderating role of demographics

**DOI:** 10.1186/s12887-025-06262-8

**Published:** 2025-11-07

**Authors:** Emma E. Berkelbach van der Sprenkel, Sanne L. Nijhof, Tessa M. T. Noijons, Elise M. van de Putte, Cornelis K. van der Ent, Louis J. Bont, Catrin Finkenauer, Gonneke W. J. M. Stevens

**Affiliations:** 1 Department of Pediatrics, Wilhelmina Children’s Hospital, University Medical Center Utrecht, Lundlaan 6, Utrecht, Utrecht 3584 EA The Netherlands; 2 Department of Interdisciplinary Social Science, Utrecht University, Utrecht, The Netherlands

**Keywords:** Adolescent, Chronic condition, Psychosocial, Functioning

## Abstract

**Background:**

This study investigated the differences in psychosocial functioning between adolescents with and without chronic conditions and examined the moderating influence of demographic characteristics.

**Methods:**

We conducted a cross-sectional study using nationally representative data from the Dutch Health Behavior in School-aged Children (HBSC) survey in 2017, which included 8,182 adolescents (aged 11–16 years), of whom 407 reported a chronic condition. Psychosocial functioning was assessed using ten indicators across three domains: wellbeing (life satisfaction, self-rated health, psychosomatic complaints, and mental health problems), social environment (sources of support and schoolwork pressure), and lifestyle (sleep problems, physical exercise, substance use, social media, and gaming). We examined differences in psychosocial functioning between adolescents with and without a chronic condition and assessed the potential moderating effects of demographic characteristics (gender, age, socioeconomic status, family structure, and migration background) on this association.

**Results:**

Across almost all indicators, adolescents with chronic conditions reported significantly lower levels of psychosocial functioning than those without chronic conditions. The effect sizes were medium to large for wellbeing indicators, and small to medium for the social environment and lifestyle indicators. For several wellbeing and social environment indicators, differences between adolescents with and without a chronic condition were more pronounced for females, older adolescents, and those from lower socioeconomic backgrounds. The differences in family support were greater among adolescents who did not live with both parents.

**Conclusions:**

Adolescents with self-reported chronic conditions experienced substantially lower levels of psychosocial functioning than those without. This highlights the need for monitoring and preventive interventions targeting specific groups to improve psychosocial functioning.

**Supplementary Information:**

The online version contains supplementary material available at 10.1186/s12887-025-06262-8.

## Introduction

While current treatments have reduced mortality for various conditions, chronic conditions (CCs) are becoming increasingly common among children and adolescents, with up to 25% of those aged 0–25 years growing up with a CC [1, 2]. Children and adolescents with CCs are at risk for developmental delays and may face more physical, social, behavioral, and emotional problems than their healthy counterparts [3–5]. Most previous studies have focused on specific conditions or aspects of psychosocial functioning. To our knowledge, no large population-based studies have examined how demographic factors moderate associations between CCs and a broad range of psychosocial functioning outcomes.

This study aims to provide a comprehensive overview of the differences in psychosocial functioning between adolescents with and without a self-reported CC, using a representative sample of adolescents. We hypothesize that adolescents with a self-reported CC report lower levels of psychosocial functioning across a broad range of indicators, as compared to adolescents without a self-reported CC, in line with previous research [4]. However, not all adolescents who grow up with a CC may face similar problems, nor do they experience them in the same way. Some research suggests that the psychosocial impact of a CC may be more substantive for adolescents who are female, older, from a lower socioeconomic background, not living with both parents, or with a migration background [3, 6–11]. To identify subgroups that may be at risk for poorer outcomes, this study investigates whether differences in psychosocial functioning between adolescents with and without a self-reported CC vary across the demographic characteristics gender, age, socio-economic status (SES), family structure, and migration background. By exploring these demographic moderators, we aim to better understand the complexity of the impact of CCs on adolescent psychosocial functioning and inform targeted, effective interventions for those most at risk.

## Methods

### Participants

This study used Dutch data from the 2017 Health Behavior in School-aged Children (HBSC) survey [12]. The HBSC survey is aimed at understanding the wellbeing and health behaviors of children aged 11 to 16 years and is carried out every four years in over 45 countries, mainly across Europe [12]. A two-stage random cluster sampling procedure was employed. A random sample of schools was drawn and stratified according to urbanization level. Next, each participating school provided a list of all classes, and two to five classes were randomly selected (depending on the school size) [12]. Participant anonymity was ensured with active consent from participants and passive consent from parents [12]. Ethical approval was granted by Utrecht University’s Faculty of Social Sciences Ethics Committee (FETC17-079).

HBSC is a school-based study in which students complete questionnaires in the classroom during school hours under supervision of trained staff. All students in the selected classes were invited to participate and were included in the sample if they met the age criteria, provided informed consent, and completed the questionnaires. Only Dutch-language questionnaires were used, as most adolescents with a migration background were born in the Netherlands and all students attended Dutch classes [12]. The questionnaires are age-appropriate, and students were able to read and complete them independently [12].

The study included 8,182 adolescents from 72 primary and 85 secondary schools with response rates of 95% and 90%, respectively. The participants’ average age was 13.87 (σ = 1.64), with 51.2% female and 78.5% native Dutch. Of the adolescents, 18% were in primary school and 82% were in secondary school. Within secondary schools, 13.8% of adolescents were in vocational, 24.5% in intermediate secondary, 20.5% in higher secondary, and 23.3% in pre-university education. Detailed sample data are presented in Table 1.

**Table 1:** Sociodemographic and individual characteristics of the study population

		Total (*n* = 8182)	No CC (*n* = 7775)	CC (*n* = 407)	*p*-value
Gender, *n* (%)					
Male	*n* = 8182	3993 (48.8%)	3796 (48.8%)	197 (48.4%)	*p* =.869
Female		4189 (51.2%)	3979 (51.2%)	210 (51.6%)	
Age, *M* (SD)	*n* = 8182	13.87 (1.64)	13.85 (1.64)	14.23 (1.56)	*p* <.001
SES, continuous, *M* (SD)	*n *= 7953	9.00 (1.86)	9.01 (1.86)	8.76 (1.89)	*p* =.013
SES, categories, *n* (%)					
Low	*n* = 7953	756 (9.5%)	700 (9.3%)	56 (14.2%)	*p* =.002
Middle		3905 (49.1%)	3712 (49.1%)	193 (49.1%)	
High		3292 (41.4%)	3148 (41.6%)	144 (36.6%)	
Family structure, *n* (%)					
Living with both parents	*n* = 8165	6302 (77.2%)	6018 (77.5%)	284 (70.3%)	*p* <.001
Not living with both parents		1863 (22.8%)	1743 (22.5%)	120 (29.7%)	
Migration background, *n* (%)					
Native Dutch	*n* = 7783	6423 (83%)	6093 (82.8%)	330 (88%)	*p* =.008
Migration background		1315 (17%)	1270 (17.2%)	45 (12%)	

*SES *Socioeconomic status, *CC *Chronic condition

### Measures

#### Chronic conditions

Participants answered the question “*Is there anyone at your home who has been physically and/or mentally ill or disabled for more than three months?*“, accompanied by examples of conditions and disabilities (e.g.,* cancer*,* diabetes*,* heart disease*,* depression*,* addiction*,* autism*,* intellectual disability*). The adolescents specified whether they or someone in their household had such a condition. Based on whether participants self-reported having a condition, they were divided into two groups: adolescents without a CC and adolescents with a CC (0 = no CC, 1 = CC). Chronic conditions were not specified by type, which precluded examination of heterogeneity across different conditions.

#### Demographic information and individual characteristics

Socio-demographic variables included gender (0 = male, 1 = female), age, SES, family structure (0 = not living with both biological parents, 1 = living with both biological parents), and migration background (0 = native Dutch, 1 = migration background). SES was assessed using the Family Affluence Scale-III (FAS-III), which is a validated measure of perceived family wealth reported by adolescents [13] It yields scores ranging from 0 to 13, thereby differentiating between low (0–7), medium (8–11), and high (12–13) affluence [14]. Adolescents with at least one parent born outside of the Netherlands were classified as having a migration background.

#### Psychosocial functioning

Three domains encompassing ten psychosocial indicators were assessed using validated scales or items: wellbeing (life satisfaction [15], self-rated health [16], psychosomatic complaints [17], and mental health problems [18]), social environment (sources of support [19] and schoolwork pressure), and lifestyle (sleep problems [20], physical exercise [21], substance use, social media [22], and gaming [23]). All composite measures have been validated for use among adolescents, with several validated specifically among adolescents with CCs, and have demonstrated good psychometric properties [15–23]. Detailed questionnaire characteristics are presented in Table 2.

**Table 2:** Psychosocial domains, corresponding items/subscales and internal consistency measurements. All measures have been validated for use among adolescents, including populations with chronic conditions [15–23]

Domain (questionnaire)	Items/ subscales used	Values and labels	Cronbach’s alpha
**WELLBEING**			
Life satisfaction [15] (Cantril ladder)	How do you feel about your life?	0 (worst possible life) to 10 (best possible life)	N/A
Self-rated health [16] (SRH)	What do you think of your own health?	0 to 4, ranging from bad; reasonable; good; excellent	N/A
Psychosomatic complaints [17] (HBSC Symptom Checklist (SCL))	Headache, stomachache, backache, feeling low, irritability or bad temper, feeling nervous, difficulties in getting to sleep, feeling dizzy, tiredness, and exhaustion (10 items) in last 6 months (mean score was used for analysis)	0 to 5, ranging from rarely or never; about every month; about every week; more than once a week; about every day	0.859
Mental health problems [18] (Strengths and Difficulties Questionnaire)	Conduct problems (5 items)	Range 0–10: higher score = more problems (recoded per subscale from items scoring not true; somewhat true; certainly true)	0.443
	Hyperactivity-inattention (5 items)		0.696
	Emotional symptoms (5 items)		0.698
	Peer relationship problems (5 items)		0.434
**SOCIAL ENVIRONMENT**			
Sources of support [19] (Multidimensional Scale of Perceived Social Support)	Support at home/family support (4 items)	Range 1–7: higher score = more support at home	0.913
	Support from friends/peer support (4 items)	Range 1–7: higher score = more support from friends	0.928
Schoolwork pressure	How much pressure do you feel from the schoolwork you have to do?	1 to 4, ranging from not at all; a little; quite a lot; a lot	N/A
**LIFESTYLE**			
Sleep problems [20] (adapted version Groningen sleep quality scale)	Sleep quality, sleep quantity, time to fall asleep, interruption and continuity (4 items)	Range: 1–5, higher score = more sleep problems	0.775
Physical exercise [21]	Over the past 7 days, on how many days were you physically active for a total of at least 60 min per day?	Range: 0–7 days	N/A
Substance use	Smoking (frequency in the last 30 days)	1 to 7, ranging from never; 1 or 2 days; 3 to 5 days; 6 to 9 days; 10 to 19 days; 20 to 29 days; 30 days (or more)	N/A
	Alcohol use (frequency in the last 30 days)		N/A
Gaming and social media [22, 23] (Social Media Disorder Scale; Internet Gaming Disorder Scale)	HBSC subscale frequency of online contact on platforms such as WhatsApp, Snapchat, Instagram, or Facebook (4 items: close friends, wider social circle, internet acquaintances, and others)	1 to 5, ranging from never or almost never; at least every week; daily or almost daily; several times each day; almost all the time throughout the day	0.673
	HBSC subscale problematic social media use (9 items)	Range: 0–9, higher score = more problematic social media use. This scale discriminates between normative (0–1), risky (2–5), and problematic (6–9) social media use.	0.697
	How often do you play games (on the computer, Xbox, PlayStation, Wii, portable game console, iPad or smartphone)?	1 to 6, ranging from (almost) never; less than 1 day per week; 1 day per week; 2–3 days per week; 4–5 days per week; (almost) every day	N/A
	HBSC subscale of problematic gaming behavior (9 items)	Range: 0–9, higher score = more problematic gaming behavior. This scale differentiates between normal (0–1), risky (2–5), and disordered gaming behaviors (6–9).	0.735

### Statistical analyses

The participants’ characteristics were summarized using descriptive statistics. Demographic differences and differences in psychosocial functioning between the groups (no CC vs. CC) were analyzed using χ2 tests for categorical data. Independent t-tests or Mann-Whitney U tests were conducted for continuous variables based on distribution normality. SES was categorized in descriptive analyses and treated as continuous in further analyses. Differences in psychosocial functioning between the groups (no CC vs. CC) were examined by separately analyzing each psychosocial indicator. We determined the effect sizes using Cohen’s d, with values of 0.2 for small effects, 0.5 for medium effects, and 0.8 for large effects, respectively.

Furthermore, separate linear regression analyses were performed to explore the association between CC and psychosocial functioning, with each psychosocial functioning indicator serving as the dependent variable. These analyses also investigated how the relationship between CC and psychosocial functioning was moderated by gender, age, SES, family structure, and migration background, while controlling for all other moderators. The interaction terms for continuous variables (age and SES) were computed using Z-scores. The reported metrics include coefficients (*B*), standard errors (*SE*), standardized regression coefficients (*β*), *t-*values, and *p-*values for the main, moderator, and interaction effects (CC × gender, age, SES, family structure, and migration background), as reported in Supplement 1.

Statistical significance was set at *p* <.05. As we repeatedly tested moderating effects on the same indicator, we applied a post-hoc Bonferroni correction for multiple comparisons, making *p*-values ≤ 0.01 significant for the regression analyses. All analyses were performed using SPSS (version 29.0; IBM, Armonk, NY, USA).

## Results

### Study population

Significant demographic differences were observed between adolescents with (*n* = 407) and without (*n* = 7,775) a CC, as shown in Table 1. Adolescents with CCs were older (14.23 ± 1.56 vs. 13.85 ± 1.64) and less likely to have a migration background (12% vs. 17.2%). Additionally, a higher percentage of those with CCs did not live with both parents (29.7% vs. 22.5%) and came from lower socioeconomic backgrounds (8.76 ± 1.89 vs. 9.01 ± 1.86).

### Differences in psychosocial functioning between adolescents with and without CC

Adolescents with a CC showed significantly poorer outcomes on most psychosocial indicators than their peers without a CC (see Table 3; *p*-values < 0.001–0.002). In terms of wellbeing, adolescents with a CC reported lower life satisfaction (*d* = 0.81), poorer self-rated health (*d* = 0.62), and more psychosomatic complaints (*d* = −0.78). Mental health problems were more prominent among adolescents with CCs, with higher levels of conduct problems (*d* = −0.46), hyperactivity-inattention (*d* = −0.45), emotional symptoms (*d* = −0.82), and peer-relationship problems (*d* = −0.64). The effect sizes ranged from medium to large, indicating substantial differences in wellbeing indicators between adolescents with and without a CC.

**Table 3:** Differences in psychosocial outcomes in adolescents with and without a self-reported chronic condition (CC)

Outcome	No CC			CC			Effect size	*p*-value
	*N*	M	SD	*N*	M	SD	Cohen’s D	
Life Satisfaction [range 0–10]	7750	7.76	1.49	407	6.51	2.28	0,81	< 0.001
Self-Rated Health [range 1–4]	7763	3.13	0.68	407	2.70	0.87	0,62	< 0.001
Psychosomatic Complaints [range 1–5]	7752	1.95	0.80	406	2.58	1.04	−0,78	< 0.001
Conduct Problems [range 0–10]	7733	1.82	1.47	403	2.50	1.91	−0,46	< 0.001
Hyperactivity-Inattention [range 0–10]	7716	4.07	2.37	402	5.14	2.46	−0,45	< 0.001
Emotional Symptoms [range 0–10]	7733	2.35	2.16	403	4.16	2.82	−0,82	< 0.001
Peer Relationship Problems [range 0–10]	7715	1.63	1.58	402	2.65	1.88	−0,64	< 0.001
Family Support [range 1–7]	7746	6.12	1.26	404	5.50	1.66	0,48	< 0.001
Peer Support [range 1–7]	7761	5.80	1.32	406	5.45	1.57	0,26	< 0.001
Schoolwork pressure [range 1–4]	7625	2.24	0.87	394	2.56	0.96	−0,37	< 0.001
Sleep Problems [range 1–5]	7699	2.67	0.97	398	3.14	1.14	−0,48	< 0.001
Physical Exercise [range 0–7]	7742	5.30	1.96	405	4.92	2.12	0,19	< 0.001
Alcohol Use [range 1–7]	7698	1.35	0.84	402	1.45	1.01	−0,12	0.030
Smoking [range 1–7]	7485	1.18	0.86	405	1.31	1.12	−0,15	< 0.001
Social media use [range 1–5]	7693	3.90	0.89	384	3.95	0.95	−0,06	0.519
Problematic social media use [range 1–9]	7760	1.42	1.73	404	1.85	2.03	−0,25	< 0.001
Gaming [range 1–6]	7733	3.62	1.99	407	3.90	2.03	−0,14	0.002
Problematic gaming [range 1–9]	7750	0.87	1.53	405	1.26	1.82	−0,25	< 0.001

Within their social environment, adolescents with a CC experienced less family support (*d* = 0.48), less peer support (*d* = 0.26), and higher schoolwork pressure (*d* = −0.37). Regarding lifestyle factors, adolescents with a CC reported more sleep problems (*d* = −0.48) and lower engagement in weekly physical exercise (*d* = 0.19). They also reported higher smoking rates (*d* = −0.15) and alcohol use (*d* = −0.12) in the past month. Although the frequency of social media use did not differ significantly (*p* =.519), they reported problematic social media use more often (*d* = −0.25), indicated a higher gaming frequency (*d* = −0.14) and more problematic gaming (*d* = −0.25). The effect sizes of these indicators ranged from small to medium.

### Moderating demographic factors in the association of CC and psychosocial functioning

**Fig. 1:**
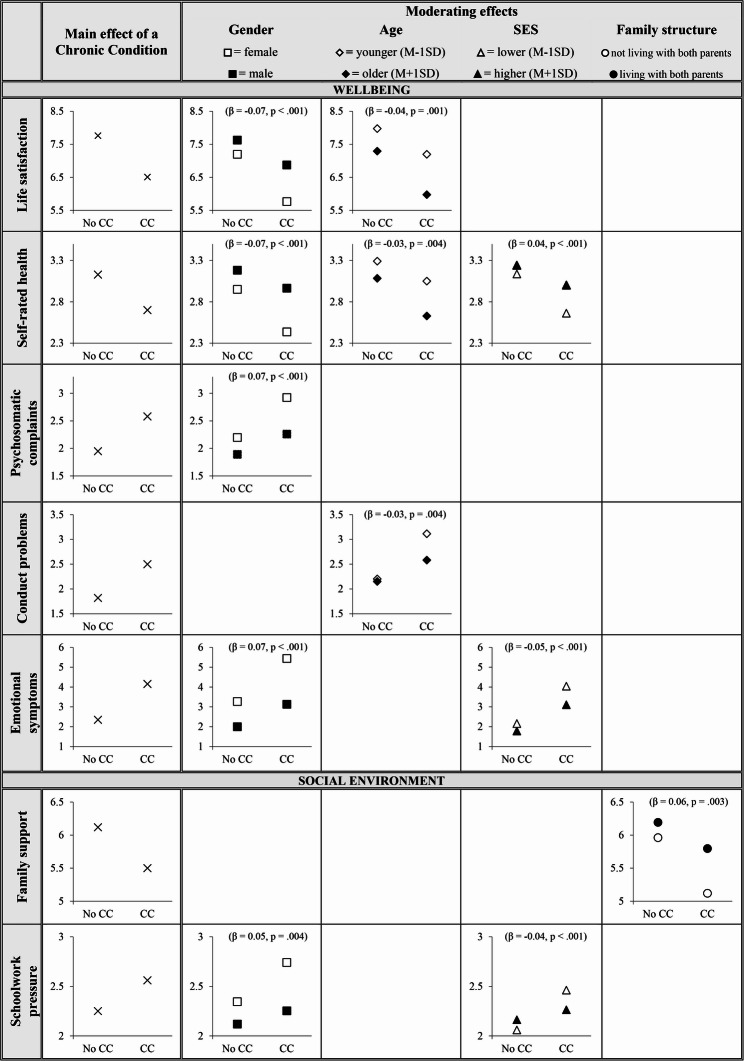
Significant interaction effects of demographic characteristics (gender, age, socioeconomic status and family structure) on the association between chronic conditions (CCs) and psychosocial functioning. Standardized regression coefficients (β) and p-values for the interaction effects are reported. Higher scores indicate poorer psychosocial functioning, except for life satisfaction, self-rated health and family support

Next, we examined whether demographic factors influenced the relationship between CCs and psychosocial indicators. Significant moderating effects are reported per demographic factor and visualized in Fig. 1. A comprehensive overview of the results is presented in Supplement 1.

We observed a significant moderating effect of gender on the association between CCs and different psychosocial indicators within the domains of wellbeing and the social environment. Differences between adolescents with and without a CC were greater for female adolescents than for male adolescents, in terms of life satisfaction (CC × gender: *B* = −0.68, *t* = −4.34, *p* <.001, 95% CI − 0.98 to − 0.37), self-rated health (CC × gender: *B* = −0.30, *t* = −4.16, *p* <.001, 95% CI − 0.44 to − 0.16), psychosomatic complaints (CC × gender: *B* = 0.36, *t* = 4.26, *p* <.001, 95% CI 0.19 to 0.52), emotional symptoms (CC × gender: *B* = 1.04, *t* = 4.65, *p* <.001, 95% CI 0.60 to 1.48), and schoolwork pressure (CC × gender: *B* = 0.26, *t* = 2.91, *p* =.004, 95% CI 0.09 to 0.44).

Age was found to significantly moderate the relationship between a self-reported CC and indicators of wellbeing. Specifically, the differences between adolescents with and without a CC were more substantive for older adolescents in terms of life satisfaction (CC × age: *B* = −0.27, *t* = −3.27, *p* =.001, 95% CI − 0.43 to − 0.11) and self-rated health (CC × age: *B* = −0.11, *t* = −2.85, *p* =.004, 95% CI − 0.18 to − 0.03), and smaller for conduct problems (CC × age: *B* = −0.24, *t* = −2.85, *p* =.004, 95% CI − 0.40 to − 0.08).

SES significantly moderated the relationship between a self-reported CC and indicators of wellbeing and social environment. Differences between a self-reported CC and no CC were greater for adolescents with lower SES in terms of self-rated health (CC × SES: *B* = 0.12, *t* = 3.31, *p* <.001, 95% CI 0.05 to 0.19), emotional symptoms (CC × SES: *B* = −0.46, *t* = −4.12, *p* <.001, 95% CI − 0.68 to − 0.24), and schoolwork pressure (CC × SES: *B* = −0.15, *t* = −3.38, *p* <.001, 95% CI − 0.24 to − 0.06).

Family structure had a significant moderating effect on the relationship between self-reported CCs and family support. Differences between a self-reported CC and no CC were greater for adolescents not living with both parents in terms of family support (CC × family structure: *B* = 0.45, *t* = 2.93, *p* =.003, 95% CI 0.15 to 0.74), as compared to those who lived with both parents.

No moderating effects of migration background were observed in the association between reporting a CC and the psychosocial indicators assessed.

## Discussion

This study aimed to investigate differences in psychosocial functioning between adolescents with and without a self-reported chronic condition (CC), focusing on how gender, age, SES, family structure, and migration background moderated these differences. The results highlighted the challenges experienced by adolescents with a self-reported CC in the domains of wellbeing, social environment, and lifestyle when compared to their peers without a CC. These findings are consistent with our previous research [4], yet extend the scope by assessing a broader range of psychosocial indicators. This study also highlighted how the impact of a self-reported CC varies across demographic groups. Differences in psychosocial functioning between adolescents with and without self-reported CCs were more pronounced across several psychosocial indicators for those who were female, older, had a lower SES, or did not live with both parents.

When compared to boys, differences between girls with and without a CC were more sizeable for five psychosocial indicators: life satisfaction, self-rated health, psychosomatic complaints, emotional symptoms, and schoolwork pressure. These findings align with existing literature [7, 8, 24], suggesting that the experience of living with a CC may differ significantly between females and males. Such disparities raise important questions regarding potential underlying causes. These may include societal influences, such as cultural norms and gender biases in healthcare, differences in coping strategies, a greater tendency among females towards internalizing problems, or biological factors and sex-differences, including hormonal changes specific to females [25–27].

The role of age as a moderator was evident in the differential impact on the wellbeing domain when comparing adolescents with and without a CC. Specifically, differences in conduct problems between adolescents with and without a CC were greater for younger adolescents, while differences in life satisfaction and self-rated health were greater for older adolescents. The behavioral problems among younger adolescents with a CC may be due to difficulties in adjusting to and comprehending their condition, feelings of isolation, or being subject to bullying [3, 28]. In contrast, lower life satisfaction and self-rated health among older adolescents with a CC may be related to an increased awareness of their condition and its cumulative impact. The physical and psychological challenges associated with living with a CC can influence the developmental milestones of adolescence, such as physical changes, identity formation, and the quest for autonomy as adolescents transition into adulthood [8, 29–31]. It may also be that the psychosocial manifestations of a CC evolve through adolescence, shifting from externalizing behaviors, such as conduct problems, to more internalizing behaviors, reflected in a decrease in life satisfaction.

Our findings also indicated a significant moderating effect of SES, with adolescents from lower SES backgrounds experiencing a greater disparity in self-rated health, emotional symptoms, and schoolwork pressure when they have a CC, compared to their peers from higher SES backgrounds. Previous studies have described that adolescents with a CC may be more likely to struggle with challenges such as increased academic demands and social stressors, which may also be exacerbated by their SES [32, 33]. Our findings are consistent with prior research that links low SES to adverse health outcomes in general [33, 34]. Additionally, lower SES has been consistently associated with increased school absenteeism and poorer school experience among adolescents with CCs [32].

Lastly, our results showed that family structure significantly moderated the association between CCs and family support. Specifically, for adolescents who did not live with both parents, differences in family support between adolescents with and without a CC were stronger than for those who lived with both parents. This lower perceived family support may be due to a variety of factors, including reduced emotional and social presence, increased caregiving responsibilities, limited availability of resources, and greater exposure to conflicts or stressors, such as financial difficulties or divorce [35]. These factors are particularly critical considering the increased support needs of adolescents who grow up with a CC [36, 37]. Adequate and consistent parental support has been shown to positively affect treatment adherence, self-management, mental wellbeing, and the overall quality of life of adolescents with CCs [36, 38, 39]. Consequently, it is important to assess and address the family’s ability to cope with the challenges they encountered in clinical settings.

This study offers insight into the psychosocial challenges faced by adolescents with self-reported CCs, including the domains of wellbeing, the social environment, and lifestyle. Our findings emphasize the importance of consistent psychosocial monitoring, along with regular biomedical evaluations. For instance, this approach encompasses the use of generic and illness-specific patient-reported outcome measures (PROMs), dialogue tools for assessing the impact of illness on adolescents’ overall health and wellbeing, and various digital tools aimed at promoting wellbeing [40]. It is essential to tailor these approaches to the adolescent’ developmental stages [40]. Moreover, demographic factors that might affect psychosocial vulnerability, such as gender, age, SES, and family structure, should be carefully considered while avoiding stereotyping and stigmatization. It is vital to ensure that professionals are equipped to address relevant psychosocial themes and are aware of the possible referral processes for appropriate care. Finally, the potential role of (collective) preventive psychosocial health interventions outside the hospital context deserves further attention.

This study had several methodological limitations that warrant consideration. First, it is important to note that our classification of self-reported CC may not align with the clinical diagnosis of CC. The observed discrepancy in prevalence, with only 5% self-reported CC in our study compared with 25% in the current Dutch healthcare data, underscores this distinction [2]. Furthermore, adolescents who self-report a CC may not always receive a formal diagnosis from a healthcare professional. Future studies should explore why adolescents with a CC do or do not self-report as such. It should be noted that the HBSC survey does not include information on the specific types of chronic conditions. Therefore, we were not able to provide an overview of the types of conditions among our sample of adolescents or to differentiate between distinct chronic conditions. This is an important limitation, as the psychosocial impact may vary across types of conditions. Additionally, although our large, population-representative sample focused on Dutch adolescents, this may restrict the generalizability of our results to other contexts. Moreover, our study found lower self-reported CCs among adolescents with an immigrant background than among native Dutch adolescents, a finding that warrants further exploration to understand its implications for the generalizability of our results regarding migration backgrounds. Finally, the cross-sectional design of our study precludes causal interpretations or tracking of changes over time.

While our study enhances our understanding of the complex relationship between CCs and psychosocial functioning in adolescents, it also emphasizes the need for further longitudinal research. Identifying risk and resilience factors can inform the development of targeted (preventive) interventions to mitigate the impact of CCs on adolescents’ psychosocial functioning.

## Conclusion

Adolescents with self-reported chronic conditions (CCs) have lower levels of psychosocial functioning across the domains of wellbeing, social environment, and lifestyle than those without self-reported CCs. Beyond highlighting the heightened risk of psychosocial problems among adolescents with CCs, this study also showed that females, older adolescents, and those with lower SES backgrounds are particularly vulnerable. These findings underscore the need for monitoring and targeted (preventive) interventions to support the diverse psychosocial needs of adolescents with CCs.

## Supplementary Information

Supplementary Material 1.

## Data Availability

The datasets used and/or analyzed during the current study are available from the corresponding author on reasonable request.

## Abbreviations

CC: Chronic condition
SES: Socio-economic status
HBSC: Health Behavior in School-aged Children
FAS-III: Family Affluence Scale-III
SRH: Self-Rated Health
HBSC-SCL: HBSC Symptom Checklist
SDQ: Strengths and Difficulties Questionnaire
MSPSS: Multidimensional Scale of Perceived Social Support
SMD-scale: Social Media Disorder scale
IGD-scale: Internet Gaming Disorder scale
