# Supplementary material for: Chronic conditions and adolescent psychosocial functioning: the moderating role of demographics
Source: BMC Pediatr. 2025 Nov 7;25:916. doi: 10.1186/s12887-025-06262-8 (PMC12595679; doi:10.1186/s12887-025-06262-8)
Supplement: Supplementary file 1 — Supplementary Material 1. [file 12887_2025_6262_MOESM1_ESM.docx]

**Supplement 1.** Main effects of a CC and demographic factors on psychosocial functioning, and their moderation effects

| **Moderator:** | **Gender ^1^** | | | | | **Age** | | | | | **SES** | | | | | **Family structure ^2^** | | | | | **Migration background ^3^** | | | | |
| --- | --- | --- | --- | --- | --- | --- | --- | --- | --- | --- | --- | --- | --- | --- | --- | --- | --- | --- | --- | --- | --- | --- | --- | --- | --- |
|  | B | SE | β | t- value | p-value | B | SE | β | t-  value | p-  value | B | SE | β | t- value | p-value | B | SE | β | t-  value | p-  value | B | SE | β | t-  value | p-  value |
| **WELLBEING** | | | | | | | | | | | | | | | | | | | | | | | | | |
| **Life satisfaction** | | | | | | | | | | | | | | | | | | | | | | | | | |
| Constant | 7.62 | .04 |  | 185.59 | < .001 | 7.64 | .04 |  | 186.49 | < .001 | 7.64 | -.04 |  | 186.41 | < .001 | 7.64 | .04 |  | 183.51 | < .001 | 7.64 | .04 |  | 186.31 | < .001 |
| Main effect CC | -.75 | .11 | -.10 | -6.74 | < .001 | -1.04 | .08 | -.14 | -13.03 | < .001 | -1.08 | .08 | -.15 | -12.75 | < .001 | -1.10 | .15 | -.15 | -7.45 | < .001 | -1.08 | .08 | -.15 | -12.97 | < .001 |
| Main effect moderator | -.43 | .03 | -.14 | -12.49 | < .001 | -.34 | .02 | -.22 | -20.01 | < .001 | .16 | .02 | .10 | 8.80 | < .001 | .44 | .05 | .12 | 10.41 | < .001 | .14 | .05 | .03 | 2.97 | .003 |
| Moderator effect | -.68 | .16 | -.07 | -4.34 | < .001 | -.27 | .08 | -.04 | -3.27 | .001 | .16 | .08 | .02 | 2.08 | .038 | -.01 | .17 | -.001 | -.05 | .962 | -.16 | .24 | -.008 | -.66 | .513 |
| **Self-rated health** | | | | | | | | | | | | | | | | | | | | | | | | | |
| Constant | 3.18 | .02 |  | 169.71 | < .001 | 3.19 | .02 |  | 170.53 | < .001 | 3.19 | .02 |  | 170.58 | < .001 | 3.19 | .02 |  | 167.91 | < .001 | 3.19 | .02 |  | 170.40 | < .001 |
| Main effect CC | -.22 | .05 | -.07 | -4.26 | < .001 | -.35 | .04 | -.11 | -9.48 | < .001 | -.36 | .04 | -.11 | -9.90 | < .001 | -.39 | .07 | -.12 | -5.86 | < .001 | -.37 | .04 | -.12 | -9.80 | < .001 |
| Main effect moderator | -.23 | .02 | -.17 | -14.81 | < .001 | -.10 | .01 | -.15 | -13.34 | < .001 | .05 | .01 | .08 | 6.62 | < .001 | .10 | .02 | .06 | 5.11 | < .001 | -.04 | .02 | -.02 | -2.03 | .042 |
| Moderator effect | -.30 | .07 | -.07 | -4.16 | < .001 | -.11 | .04 | -.03 | -2.85 | .004 | .12 | .04 | .04 | 3.31 | < .001 | .03 | .08 | .01 | .41 | .682 | -.03 | .11 | .003 | .25 | .805 |
| **Psychosomatic complaints** | | | | | | | | | | | | | | | | | | | | | | | | | |
| Constant | 1.89 | .02 |  | 85.95 | < .001 | 1.89 | .02 |  | 85.80 | < .001 | 1.88 | .02 |  | 85.78 | < .001 | 1.88 | .02 |  | 84.29 | < .001 | 1.89 | .02 |  | 85.79 | < .001 |
| Main effect CC | .37 | .06 | .10 | 6.15 | < .001 | .54 | .04 | .14 | 12.56 | < .001 | .54 | .04 | .14 | 12.86 | < .001 | .63 | .08 | .17 | 8.02 | < .001 | .54 | .05 | .14 | 12.14 | < .001 |
| Main effect moderator | .31 | .02 | .19 | 16.66 | < .001 | .12 | .01 | .15 | 13.33 | < .001 | -.02 | .01 | -.02 | -1.58 | .114 | -.14 | .02 | -.07 | -6.40 | < .001 | .09 | .03 | .04 | 3.44 | < .001 |
| Moderator effect | .36 | .08 | .07 | 4.26 | < .001 | .06 | .04 | .02 | 1.30 | .194 | -.08 | .04 | -.02 | -1.82 | .069 | -.11 | .09 | -.03 | -1.20 | .231 | .07 | .13 | .007 | .58 | .560 |
| **Conduct problems** | | | | | | | | | | | | | | | | | | | | | | | | | |
| Constant | 2.17 | .04 |  | 51.74 | < .001 | 2.18 | .04 |  | 52.10 | < .001 | 2.18 | .04 |  | 52.09 | < .001 | 2.18 | .03 |  | 51.40 | < .001 | 2.18 | .04 |  | 52.07 | < .001 |
| Main effect CC | .79 | .11 | .11 | 6.90 | < .001 | .67 | .08 | .10 | 8.24 | < .001 | .64 | .08 | .09 | 7.90 | < .001 | .53 | .15 | .08 | 3.54 | < .001 | .60 | .09 | .09 | 7.05 | < .001 |
| Main effect moderator | -.31 | .04 | -.10 | 8.82 | < .001 | -.03 | .02 | -.02 | -1.50 | .135 | -.03 | .02 | -.02 | -1.75 | .080 | -.32 | .04 | -.09 | -7.57 | < .001 | .33 | .05 | .08 | 7.03 | < .001 |
| Moderator effect | -.33 | .16 | -.03 | -2.04 | .041 | -.24 | .08 | -.03 | -2.85 | .004 | .11 | .08 | .02 | 1.37 | .170 | .13 | .18 | .02 | .71 | .479 | .17 | .24 | .009 | .70 | .482 |
| **Hyperactivity-inattention** | | | | | | | | | | | | | | | | | | | | | | | | | |
| Constant | 4.62 | .07 |  | 69.00 | < .001 | 4.62 | .07 |  | 69.30 | < .001 | 4.62 | .07 |  | 69.31 | < .001 | 4.62 | .07 |  | 68.23 | < .001 | 4.62 | .07 |  | 69.32 | < .001 |
| Main effect CC | .95 | .18 | .09 | 5.22 | < .001 | .93 | .12 | .08 | 7.14 | < .001 | .91 | .13 | .08 | 7.09 | < .001 | .88 | .24 | .08 | 3.66 | < .001 | .79 | .14 | .07 | 5.84 | < .001 |
| Main effect moderator | -.11 | .06 | -.02 | -1.95 | .051 | .04 | .93 | .02 | 1.34 | .181 | .04 | .03 | .02 | 1.51 | .131 | -.50 | .07 | -.09 | -7.36 | < .001 | -.58 | -.58 | -.09 | -7.74 | < .001 |
| Moderator effect | -.14 | .25 | -.01 | -.54 | .589 | -.23 | .13 | -.02 | -1.75 | .080 | .22 | .13 | .02 | 1.75 | .080 | .005 | .28 | .00 | .02 | .986 | .71 | .71 | .02 | 1.84 | .065 |
| **Emotional symptoms** | | | | | | | | | | | | | | | | | | | | | | | | | |
| Constant | 1.20 | .06 |  | 33.90 | < .001 | 1.98 | .06 |  | 33.58 | < .001 | 1.97 | .06 |  | 33.56 | < .001 | 1.96 | .06 |  | 32.85 | < .001 | 1.98 | .06 |  | 33.58 | < .001 |
| Main effect CC | 1.13 | .16 | .11 | 7.04 | < .001 | 1.61 | .12 | .15 | 13.95 | < .001 | 1.61 | .11 | .15 | 14.25 | < .001 | 1.90 | .21 | .18 | 8.99 | < .001 | 1.66 | .12 | .16 | 13.85 | < .001 |
| Main effect moderator | 1.27 | .05 | .28 | 25.88 | < .001 | .19 | .03 | .08 | 7.66 | < .001 | -.14 | .03 | -.06 | -5.45 | < .001 | -.34 | .06 | .06 | -5.64 | < .001 | -.16 | .07 | -.03 | -2.44 | .015 |
| Moderator effect | 1.04 | .22 | .07 | 4.65 | < .001 | .28 | .12 | .03 | 2.35 | .019 | -.46 | .11 | -.05 | -4.12 | < .001 | -.33 | .25 | .25 | -1.30 | .192 | .05 | .33 | .002 | .14 | .891 |
| **Peer relationship problems** | | | | | | | | | | | | | | | | | | | | | | | | | |
| Constant | 1.79 | .04 |  | 40.24 | <.001 | 1.79 | .04 |  | 40.30 | < .001 | 1.79 | .04 |  | 40.29 | < .001 | 1.80 | .05 |  | 39.96 | < .001 | 1.79 | .04 |  | 40.29 | < .001 |
| Main effect CC | .88 | .12 | .12 | 7.24 | <.001 | .92 | .09 | .12 | 10.55 | < .001 | .95 | .09 | .13 | 11.15 | < .001 | .75 | .16 | .10 | 4.71 | < .001 | .97 | .09 | .13 | 10.67 | < .001 |
| Main effect moderator | -.13 | .04 | -.04 | -3.54 | <.001 | .003 | .02 | .002 | .18 | .857 | -.20 | .02 | -.12 | -10.41 | < .001 | -.21 | .05 | -.05 | -4.63 | < .001 | .36 | .05 | .08 | 7.14 | < .001 |
| Moderator effect | .15 | .17 | .02 | .90 | .366 | .19 | .09 | .03 | 2.12 | .034 | -.05 | .08 | -.01 | -.59 | .553 | .29 | .19 | .03 | 1.53 | .126 | -.07 | .26 | -.003 | -.25 | .801 |
| **SOCIAL ENVIRONMENT** | | | | | | | | | | | | | | | | | | | | | | | | | |
| **Family support** | | | | | | | | | | | | | | | | | | | | | | | | | |
| Constant | 5.94 | .04 |  | 165.61 | < .001 | 5.94 | .04 |  | 166.29 | < .001 | 5.94 | .04 |  | 166.24 | < .001 | 5.96 | .04 |  | 164.33 | <.001 | 5.94 | .04 |  | 166.20 | < .001 |
| Main effect CC | -.46 | .10 | -.08 | -4.67 | < .001 | -.48 | .07 | -.08 | -6.90 | < .001 | -.51 | .07 | -.09 | -7.41 | < .001 | -.84 | .13 | -.14 | -6.54 | <.001 | -.46 | .07 | -.08 | -6.34 | < .001 |
| Main effect moderator | .01 | .03 | .01 | .39 | .649 | -.16 | .02 | -.12 | -10.51 | < .001 | .08 | .02 | .06 | 4.86 | < .001 | .23 | .04 | .08 | 6.38 | <.001 | -.10 | .04 | -.03 | -2.55 | .011 |
| Moderator effect | -.12 | .14 | -.02 | -.91 | .365 | -.18 | .07 | -.03 | -2.52 | .012 | .09 | .07 | .02 | 1.39 | .166 | .45 | .15 | .06 | 2.93 | .003 | -.48 | .21 | -.03 | -2.30 | .021 |
| **Peer support** | | | | | | | | | | | | | | | | | | | | | | | | | |
| Constant | 5.49 | .04 |  | 148.40 | < .001 | 5.50 | .04 |  | 148.99 | < .001 | 5.50 | .04 |  | 148.98 | < .001 | 5.49 | .04 |  | 146.56 | < .001 | 5.49 | .04 |  | 148.95 | < .001 |
| Main effect CC | -.24 | .10 | -.04 | -2.38 | .017 | -.35 | .07 | -.06 | -4.88 | < .001 | -.34 | .07 | -.05 | -4.77 | < .001 | -.21 | .12 | -.03 | -1.56 | .119 | -.32 | .08 | -.05 | -4.25 | < .001 |
| Main effect moderator | .59 | .03 | .22 | 19.16 | < .001 | -.04 | .02 | -.03 | -2.56 | .011 | .10 | .02 | .08 | 6.29 | < .001 | .05 | .04 | .01 | 1.19 | .234 | -.09 | .04 | -.03 | -2.23 | .026 |
| Moderator effect | -.20 | .14 | -.02 | -1.41 | .158 | .04 | .07 | .007 | .58 | .563 | .04 | .07 | .01 | .52 | .604 | -.19 | .16 | -.03 | -1.20 | .231 | -.19 | .21 | -.01 | -.88 | .381 |
| **Schoolwork pressure** | | | | | | | | | | | | | | | | | | | | | | | | | |
| Constant | 2.12 | .02 |  | 89.46 | < .001 | 2.11 | .02 |  | 89.48 | < .001 | 2.11 | .02 |  | 89.49 | < .001 | 2.11 | .02 |  | 88.14 | < .001 | 2.11 | .02 |  | 89.52 | <.001 |
| Main effect CC | .13 | .07 | .03 | 2.08 | .038 | .28 | .05 | .07 | 5.96 | < .001 | .25 | .05 | .06 | 5.51 | < .001 | .26 | .09 | .06 | 3.03 | .002 | .24 | .05 | .06 | 4.91 | <.001 |
| Main effect moderator | .23 | .02 | .12 | 11.41 | < .001 | .22 | .01 | .26 | 22.78 | < .001 | .05 | .01 | .06 | 5.15 | < .001 | -.02 | .02 | -.01 | -.84 | .399 | .05 | .03 | .02 | 2.01 | .044 |
| Moderator effect | .26 | .09 | .05 | 2.91 | .004 | -.03 | .05 | -.01 | -.59 | .555 | -.15 | .05 | -.04 | -3.38 | < .001 | .02 | .10 | .004 | .18 | .859 | .27 | .27 | .02 | 1.97 | .049 |
| **LIFESTYLE** | | | | | | | | | | | | | | | | | | | | | | | | | |
| **Sleep problems** | | | | | | | | | | | | | | | | | | | | | | | | | |
| Constant | 2.63 | .03 |  | 96.35 | < .001 | 2.62 | .03 |  | 96.48 | < .001 | 2.62 | .03 |  | 96.48 | < .001 | 2.62 | .03 |  | 95.09 | < .001 | 2.62 | .03 |  | 96.46 | < .001 |
| Main effect CC | .29 | .07 | .06 | 3.84 | < .001 | .38 | .05 | .08 | 7.17 | < .001 | .40 | .05 | .09 | 7.73 | < .001 | .36 | .10 | .08 | 3.70 | < .001 | .40 | .06 | .09 | 7.14 | < .001 |
| Main effect moderator | .30 | .02 | .15 | 13.08 | < .001 | .07 | .01 | .07 | 6.25 | < .001 | -.02 | .01 | -.03 | -2.09 | .037 | -.12 | .03 | -.05 | -4.47 | < .001 | -.06 | .03 | -.02 | -2.08 | .037 |
| Moderator effect | .22 | .10 | .04 | 2.11 | .035 | .08 | .06 | .02 | 1.40 | .163 | .05 | .05 | .01 | .97 | .973 | .05 | .12 | .01 | .44 | .663 | .02 | .16 | .002 | .13 | .133 |
| **Physical exercise** | | | | | | | | | | | | | | | | | | | | | | | | | |
| Constant | 5.42 | .06 |  | 99.05 | < .001 | 5.41 | .06 |  | 99.24 | < .001 | 5.41 | .06 |  | 99.22 | < .001 | 5.42 | .06 |  | 97.98 | < .001 | 5.41 | .06 |  | 99.24 | < .001 |
| Main effect CC | -.54 | .15 | -.06 | -3.60 | < .001 | -.39 | .11 | -.04 | -3.63 | < .001 | -.39 | .11 | -.04 | -3.74 | < .001 | -.60 | .20 | -.07 | -3.03 | .002 | -.41 | .11 | -.04 | -3.68 | < .001 |
| Main effect moderator | -.35 | .05 | -.09 | -7.77 | < .001 | -.12 | .02 | -.06 | -5.27 | < .001 | .26 | .02 | .13 | 11.08 | < .001 | .19 | .06 | .04 | 3.34 | < .001 | -.50 | .06 | -.09 | -8.07 | < .001 |
| Moderator effect | .33 | .21 | .03 | 1.57 | .116 | .09 | .11 | .01 | .79 | .432 | -.18 | .10 | -.02 | -1.78 | .075 | .31 | .23 | .03 | 1.36 | .174 | .32 | .32 | .01 | 1.03 | .305 |
| **Alcohol use** | | | | | | | | | | | | | | | | | | | | | | | | | |
| Constant | 1.52 | .02 |  | 67.93 | < .001 | 1.52 | .02 |  | 68.16 | < .001 | 1.52 | .02 |  | 68.15 | < .001 | 1.51 | .02 |  | 66.82 | < .001 | 1.52 | .02 |  | 68.15 | < .001 |
| Main effect CC | .001 | .06 | .00 | .01 | .992 | .01 | .04 | .002 | .21 | .837 | .004 | .04 | .001 | .10 | .921 | .12 | .08 | .03 | 1.50 | .133 | .003 | .05 | .001 | .06 | .952 |
| Main effect moderator | -.10 | .02 | -.06 | -5.11 | < .001 | .34 | .01 | .40 | 36.83 | < .001 | .05 | .01 | .05 | 4.71 | < .001 | -.10 | .02 | -.05 | -4.23 | < .001 | -.22 | .03 | -.10 | -8.87 | < .001 |
| Moderator effect | .01 | .09 | .001 | .06 | .950 | -.03 | .05 | -.01 | -.55 | .582 | .01 | .04 | .002 | .16 | .872 | -.16 | .09 | -.03 | -1.73 | .084 | -.01 | .13 | .00 | .04 | .969 |
| **Smoking** | | | | | | | | | | | | | | | | | | | | | | | | | |
| Constant | 1.35 | .03 |  | 54.93 | < .001 | 1.35 | .02 |  | 55.21 | < .001 | 1.35 | .02 |  | 55.22 | < .001 | 1.36 | .03 |  | 54.64 | < .001 | 1.35 | .02 |  | 55.18 | < .001 |
| Main effect CC | .14 | .07 | .03 | 2.09 | .037 | .08 | .05 | .02 | 1.64 | .101 | .09 | .05 | .02 | 2.01 | .045 | -.02 | .09 | -.01 | -.28 | .780 | .12 | .05 | .03 | 2.43 | .015 |
| Main effect moderator | -.06 | .02 | -.04 | -3.01 | .003 | .18 | .01 | .21 | 17.75 | < .001 | -.00 | .01 | _.00 | -.34 | .737 | -.17 | .03 | -.08 | -6.89 | < .001 | -.05 | .03 | -.02 | -1.93 | .054 |
| Moderator effect | -.10 | .09 | -.02 | -1.06 | .291 | .05 | .05 | .01 | .98 | .326 | .04 | .05 | .01 | .84 | .401 | .16 | .10 | .03 | 1.54 | .125 | -.26 | .14 | -.02 | -1.81 | .070 |
| **Social media use** | | | | | | | | | | | | | | | | | | | | | | | | | |
| Constant | 3.87 | .03 |  | 154.51 | < .001 | 3.86 | .03 |  | 155.01 | < .001 | 3.86 | .03 |  | 154.97 | < .001 | 3.862 | .03 |  | 152.57 | < .001 | 3.86 | .03 |  | 154.98 | < .001 |
| Main effect CC | -.07 | .07 | -.02 | -.93 | .353 | -.01 | .05 | -.003 | -.25 | .805 | -.02 | .05 | -.01 | -.46 | .648 | .003 | .09 | .001 | .03 | .976 | .01 | .05 | .002 | .13 | .898 |
| Main effect moderator | .16 | .02 | .09 | 7.56 | < .001 | .15 | .01 | .17 | 14.32 | < .001 | .08 | .01 | .09 | 7.54 | < .001 | -.10 | .03 | -.05 | -4.11 | < .001 | .18 | .03 | .07 | 6.20 | < .001 |
| Moderator effect | .08 | .10 | .02 | .87 | .386 | -.04 | .05 | -.01 | -.69 | .493 | -.01 | .05 | -.003 | -.27 | .786 | -.03 | .11 | -.01 | -.31 | .755 | -.24 | .15 | -.02 | -1.57 | .117 |
| **Problematic social media use** | | | | | | | | | | | | | | | | | | | | | | | | | |
| Constant | 1.47 | .05 |  | 29.74 | < .001 | 1.47 | .05 |  | 29.80 | < .001 | 1.47 | .05 |  | 29.79 | <.001 | 1.47 | .05 |  | 29.36 | < .001 | 1.47 | .05 |  | 29.84 | < .001 |
| Main effect CC | .28 | .14 | .04 | 2.09 | .036 | .39 | .10 | .05 | 4.05 | < .001 | .36 | .10 | .04 | 3.75 | <.001 | .33 | .18 | .04 | 1.87 | .062 | .26 | .10 | .03 | 2.63 | .009 |
| Main effect moderator | .32 | .04 | .09 | 7.71 | < .001 | .14 | .02 | .08 | 6.95 | < .001 | -.003 | .02 | -.002 | -.15 | .878 | -.33 | .05 | -.08 | -6.53 | < .001 | .23 | .06 | .05 | 4.09 | < .001 |
| Moderator effect | .12 | .19 | .01 | .66 | .511 | -.20 | .10 | -.02 | -1.95 | 0.51 | .06 | .09 | .01 | .67 | .505 | .02 | .21 | .002 | .11 | .910 | .67 | .28 | .03 | 2.36 | .018 |
| **Gaming** | | | | | | | | | | | | | | | | | | | | | | | | | |
| Constant | 4.80 | .05 |  | 102.81 | < .001 | 4.80 | .05 |  | 102.93 | < .001 | 4.80 | .05 |  | 102.96 | < .001 | 4.80 | .05 |  | 101.51 | < .001 | 4.80 | .05 |  | 102.92 | < .001 |
| Main effect CC | .12 | .13 | .01 | .94 | .350 | .35 | .09 | .04 | 3.79 | < .001 | .35 | .09 | .04 | 3.93 | < .001 | .22 | .17 | .02 | 1.33 | .183 | .31 | .10 | .03 | 3.30 | < .001 |
| Main effect moderator | -2.09 | .03 | -.53 | -53.70 | <.001 | -.42 | .02 | -.21 | -21.58 | < .001 | -.01 | .02 | -.003 | -.28 | .777 | -.09 | .05 | -.02 | -1.83 | .067 | .26 | .05 | -.05 | -4.89 | < .001 |
| Moderator effect | .42 | .18 | .03 | 2.35 | .019 | -.06 | -.09 | -.01 | -.61 | .542 | .15 | .09 | .02 | 1.65 | .099 | .15 | .20 | -.01 | .78 | .436 | .17 | .27 | .01 | .61 | .541 |
| **Problematic gaming** | | | | | | | | | | | | | | | | | | | | | | | | | |
| Constant | 1.60 | .04 |  | 39.20 | < .001 | 1.60 | .04 |  | 39.44 | <.001 | 1.60 | .04 |  | 39.46 | < .001 | 1.59 | .04 |  | 38.66 | < .001 | 1.60 | .04 |  | 39.43 | < .001 |
| Main effect CC | .50 | .11 | .07 | 4.47 | < .001 | .44 | .08 | .06 | 5.51 | <.001 | .42 | .08 | .06 | 5.37 | < .001 | .52 | .15 | .07 | 3.59 | < .001 | .39 | .08 | .05 | 4.71 | < .001 |
| Main effect moderator | -1.16 | .03 | -.37 | -34.15 | < .001 | -.05 | .02 | -.03 | -2.99 | .003 | -.04 | .02 | -.02 | -2.09 | .037 | -.17 | .17 | -.05 | -4.07 | < .001 | .01 | .05 | .003 | .26 | .794 |
| Moderator effect | -.18 | .16 | -.02 | -1.19 | .233 | -.16 | .08 | -.02 | -1.98 | .048 | .14 | .08 | .02 | 1.76 | .079 | -.17 | .02 | -.02 | -.98 | .327 | .10 | .23 | .01 | .43 | .668 |

^1^ Gender (male = 0, female =1); ^2^ Family structure (not living with both parents = 0, living with both parents =1); ^3^ Migration background (native Dutch = 0, immigration background =1).
